# NF-kappaB mediates the survival of human bronchial epithelial cells exposed to cigarette smoke extract

**DOI:** 10.1186/1465-9921-9-66

**Published:** 2008-09-23

**Authors:** Xiangde Liu, Shinsaku Togo, Mona Al-Mugotir, Huijung Kim, QiuHong Fang, Tetsu Kobayashi, XingQi Wang, Lijun Mao, Peter Bitterman, Stephen Rennard

**Affiliations:** 1 Pulmonary, Critical Care and Sleep Medicine, Department of Internal Medicine, University of Nebraska Medical Center, Omaha, Nebraska, USA; 2 Juntendo University, School of Medicine, Tokyo, Japan; 3 Won Kwang University, Kunpo Medical Center, Seoul, Republic of Korea; 4 Beijing Shijitan Hospital, Peking University, Beijing, PR China; 5 Mie University of Graduate School of Medicine, Tsu City, Japan; 6 The 3^rd ^Hospital of Peking University, Beijing, PR China; 7 University of Minnesota, Minneapolis, Minnesota, USA

## Abstract

**Background:**

We have previously reported that low concentrations of cigarette smoke extract induce DNA damage without leading to apoptosis or necrosis in human bronchial epithelial cells (HBECs), and that IL-6/STAT3 signaling contributes to the cell survival. Since NF-κB is also involved in regulating apoptosis and cell survival, the current study was designed to investigate the role of NF-κB in mediating cell survival in response to cigarette smoke exposure in HBECs.

**Methods:**

Both the pharmacologic inhibitor of NF-κB, curcumin, and RNA interference targeting p65 were used to block NF-κB signaling in HBECs. Apoptosis and cell survival were then assessed by various methods including COMET assay, LIVE/DEAD Cytotoxicity/Viability assay and colony formation assay.

**Results:**

Cigarette smoke extract (CSE) caused DNA damage and cell cycle arrest in S phase without leading to apoptosis in HBECs as evidenced by TUNEL assay, COMET assay and DNA content assay. CSE stimulated NF-κB -DNA binding activity and up-regulated Bcl-XL protein in HBECs. Inhibition of NF-κB by the pharmacologic inhibitor curcumin (20 μM) or suppression of p65 by siRNA resulted in a significant increase in cell death in response to cigarette smoke exposure. Furthermore, cells lacking p65 were incapable of forming cellular colonies when these cells were exposed to CSE, while they behaved normally in the regular culture medium.

**Conclusion:**

The current study demonstrates that CSE activates NF-κB and up-regulates Bcl-XL through NF-kB activation in HBECs, and that CSE induces cell death in cells lacking p65. These results suggest that activation of NF-κB regulates cell survival following DNA damage by cigarette smoke in human bronchial epithelial cells.

## Background

Cigarette smoke is the major preventable risk factor for a variety of diseases including lung cancer and emphysema. However, the mechanisms by which cigarette smoke causes these varying diseases are not yet fully understood. One key mechanism is believed to be the ability of cigarette smoke to damage cellular DNA leading to the accumulation of somatic cell mutations, which is believed to contribute to the development of cancer and possible chronic obstructive pulmonary disease as well [1]. In this regard, cells can protect genomic integrity by activating pathways of apoptosis in response to DNA damage. Cigarette smoke-induced DNA damage, however, does not always lead to apoptosis or necrosis [2,3]. In this content, DNA repair can also be activated following DNA damage, and the complex mechanisms that regulate whether DNA damage leads to DNA repair or apoptosis have been the subject of many recent studies [4-6].

We have previously reported that cigarette smoke induces DNA damage in human bronchial epithelial cells and lung fibroblasts without leading to apoptosis or necrosis [2,3]. These cells repaired DNA damage and were capable of proliferating and forming colonies when they were subsequently sub-cultured in normal medium. Several signaling pathways may be involved in modulating cell survival or apoptosis in response to cigarette smoke-induced DNA damage. Among these, NF-κB has been reported to play an important role in mediating cell survival. In addition, NF-κB is activated by cigarette smoke *in vitro *in cell culture and *in vivo *in animal models of cigarette smoke exposure [7,8]. Thus, the current study was designed to investigate the role of NF-κB in modulating cell survival in human bronchial epithelial cells exposed to cigarette smoke extract. We demonstrated that cigarette smoke extract at low concentration (5 and 10%) stimulated NF-κB -DNA binding activity and up-regulated Bcl-XL in HBECs. Inhibition of NF-κB by curcumin or suppression of p65 by siRNA had no effect on control cell viability, but resulted in increased cell death in cells exposed to cigarette smoke extract. These results indicate that activation of NF-κB following DNA damage plays an important role in mediating whether cell survival or apoptosis ensues.

## Methods

### Cell culture

The human bronchial epithelial cell line, BEAS-2B, was obtained from the American Type Culture Collection (CRL-9609; Rockville, MD). Normal human bronchial epithelial cells (HBECs) were acquired from bronchial biopsies of smokers with normal lung function using a previously published method with modifications [9] under a protocol approved by the University of Nebraska Human Studies Committee. Both BEAS-2B and HBEC cells were cultured in 100 mm tissue culture dishes (Falcon; BD Bioscience Discovery Labware, Bedford, MA), which were coated with 0.03 mg/ml collagen (Vitrogen 100, Angiotech BioMaterials, Palo Alto, CA), in a 1:1 mixture of LHC-9/RPMI 1640 [10,11]. Cells were passaged once weekly at a 1:3 ratio. HBECs between the 3rd and 10th passage were used for experiments.

### Antibodies for immunoblot

Following primary antibodies were purchased from Cell Signaling Technology, Inc. (Danvers, MA): anti-Stat3, anti-p65, anti-p50/p105, anti-Bcl-XL, anti-Bad, anti-Bax, anti-Bcl2, and anti-XIAP antibodies. Anti-β-actin antibody (A5441) was purchased from Sigma (St. Louise, MO).

### Cigarette smoke extract (CSE) preparation

Cigarette smoke extract (CSE) was prepared with a modification of the method of Carp and Janoff [12]. Briefly, one 100 mm long cigarette without filter (R1 Research Grade Cigarette, University of Kentucky; these were obtained prior to their being discontinued by the manufacturer and were stored (4°C) until use.) was combusted with a Mini-Pump Variable Flow (Fisher Scientific, Pittsburgh, PA). The smoke was bubbled through 25 ml double distilled water (ddH2O) at a speed of 70 cc/minute till the unburned butt is less than 1 cm long. The resulting suspension was in yellowish color and the optical density (OD) value at 405 nm wave-length was 0.120 ± 0.020. This solution was filtered through a 0.22 μm pore filter (Lida Manufacturing Corp., Kenosha, WI) to remove bacteria and large particles. This filtered solution was considered to be 100% CSE and diluted with LHC-D/RPMI 1640 medium [10,11] within 30 minutes of preparation to obtain the desired concentration used in each experiment. The LHC-D/RPMI 1640 medium was a 1:1 mixture of LHC-D and RPMI 1640, which contained no serum or growth factors.

### Quantitative TUNEL assay

DNA strand breaks were evaluated quantitatively with a colorimetric kit (Titer TACS, Trevigen, Gaithersburg, CA) that utilizes TUNEL stain in a 96-well format, following the manufacturer's instructions. Briefly, cells were cultured in 96-well plates till sub-confluent and treated with desired concentrations of cigarette smoke extract. Cells were then fixed with 3.7% Buffered Formaldehyde for 5 minutes followed by washing once with PBS. The cells were post-fixed with 100% methanol for 20 minutes and washed once with PBS. Cells were then labeled by TUNEL following the manufacturer's instructions. Absorbance at 450 nm was measured with a Benchmark microplate reader (Bio-Rad, Hercules, CA). For comparison, data are expressed as % of Positive Control (DNAse treated cells), according to the formula: (Sample OD value-blank)/(Positive Control OD value-blank) × 100.

### DNA content profiling by FACS analysis

To determine the presence of apoptotic cells, DNA content was measured by flow cytometry as reported previously [2]. Cells were cultured in 6-well plates till confluent. After treatment with CSE or as a positive control, the DNA topoisomerase inhibitor camptothecin (CPT), medium was harvested to collect floating cells and attached cells were detached from the tissue culture dishes with trypsin/EDTA. Both floating and attached cells were then pelleted together and fixed with 70% ethanol at 4°C for 30 minutes. After staining with propidium iodide (50 μg/10^6 ^cells, Sigma, St. Louis, MO), DNA content/cell cycle analysis was performed by flow cytometry. Cells with less DNA content than that of G1 cells (sub-G1 peaks or A_0 _cells) were considered apoptotic.

### COMET assay

Comet assay was performed using the CometAssay™ Kit (Trevigen, Inc. Gaithersburg, MD). Briefly, cells were cultured in 6-well plates and treated with cigarette smoke extract or camptothecin as described above. Floating and attached cells were harvested, pelleted and re-suspended with cold PBS at 10^5 ^cells/ml. The resulting cell suspension (50 μL) was mixed with 500 μL of LMAgarose and 75 μL of the agarose/cells was pipetted over the sample area of the COMET Slides. The samples were lysed, electrophoresed and stained following the manufacturer's instructions. Cells were then viewed with an epifluorescence microscopy (NIKON Eclipse E800) and photographed with a digital camera (OPTRONICS) under 200× magnification. Apoptotic index = number of cells with small DNA head with fan-like tail/total cell number × 100%.

### Immunoblots

Cells were lysed with lysing buffer (50 mM Tris buffer, pH 7.4, containing 10 mM EDTA, 2 mM EGTA, 2 mM benzamidine, 2.5 mM dithiothreitol, 2 μg/ml soybean trypsin inhibitor, 100 μM tosyl-L-lysine chloromethyl ketone, 200 μM leupeptin, and 50 μM phenylmethylsulphonyl fluoride). The cell lysate was centrifuged at 12,000 g for 10 minutes at 4°C and the precipitates were discarded. Protein concentrations in supernatants were determined by a protein dye-binding assay (Bio-Rad, Hercules, CA). Proteins were then subjected to immunoblot analysis. After heating for 3 minutes at 95°C, 10 μg of total protein was mixed with 2× sample buffer (0.5 M Tris-HCL, pH 6.8, 10% SDS, 0.1% bromphenol blue, 20% glycerol, 2% b-mercaptoethanol) and loaded into each well before performing electrophoresis with the Mini-protein 3 Cell System^® ^(Bio-Rad, Hercules, CA). The proteins were transferred to PVDF membranes (Bio-Rad, Hercules, CA) in transfer buffer (20 mM Tris, pH 8.0, 150 mM glycine, 20% methanol) at 20 V for 40 minutes with the semi-dry electrophoretic transfer system (Bio-Rad, Hercules, CA). The membrane was blocked with 5% dry-milk in PBS-Tween at room temperature for 1 hour and then exposed to primary antibodies at 4°C overnight. Target proteins were subsequently detected using horseradish peroxidase conjugated IgG with an enhanced chemiluminescence detection system (ECL, Amersham Pharmacia Biotech, Little Chalfont, Buckinghamshire, England).

### Electrophoretic mobility shift assay (EMSA)

Electrophoretic mobility shift assay (EMSA) was performed with a kit (Panomics, Inc, Redwood city, CA) following the manufacturer's instructions. Briefly, nuclear extract (5 μg) was incubated with 10 ng biotin-labeled NF-κB (p65) probe. For the "cold probe" assay, 20 ng of unlabeled (cold) NF-kB probe was mixed with sample 5 minuets before adding 10 ng biotin-labeled NF-kB probe (cold vs labeled probe ratio was 2:1). Protein-DNA complexes were then resolved by non-denaturing polyacrylamide gel electrophoresis (PAGE). After transfer to Pall Biodyne B^® ^membrane (Pall Corporation, East Hills, NY), proteins were immobilized in UV cross-linker. After blocking, avidin-HRP was applied and detected by enhanced chemiluminescence (ECL, Amersham).

### RNA interference

Targeting p65 siRNA and non-targeting control siRNA (Dharmcon, Inc, Lafayette, CO) were introduced into the cells using a method previously described with modification [13]. Briefly, normal human bronchial epithelial cells were plated into V30-coated 60 mm dishes at a density of 10^6 ^/dish so that the cells were 60–70% confluent after 1–2 days. At that time, after washing with PBS, cells were treated with Lipofectamine 2000 (Invitrogen, Carlsbad, CA) containing p65-siRNA, STAT3-siRNA or non-targeting control siRNA (final concentration 100 nM in Opti-MEM) for 6 hours followed by re-feeding with LHC-9/RPMI medium. On the next day, cells were trypsinzed and counted. One half million cells were used to examine the silencing effect. The remainder of the cells were plated into 6-well plates and exposed to cigarette smoke on the next day.

### LIVE/DEAD staining

Cell viability was evaluated by ethidium homodimer-1 dye exclusion using the LIVE/DEAD Kit, following the manufacturer's instructions (Invitrogen, Carlsbad, CA). Briefly, cells were detached by trypsinization and incubated in LHC-D/RPMI medium containing Calcein AM (Green) and ethidium homodimer-1 (EthD-1, Red) at 37°C for 15 minutes. Cells were then spun onto slides by cytospin followed by observation under fluorescence microscopy within 24 hrs. Nuclei stained by EthD-1, which appeared red, were counted as dead cells. Cell number was counted in 5 randomly chosen fields and expressed as percent of dead cells (number of red nuclear stained cells/total cell number). In general, at least 300 cells were counted.

### Clonogenic assay

Assay of clongenic growth was performed with a modification of the previously reported methods [14]. Briefly, cells were cultured in 6-well plates and treated with CSE or camptothecin for 24 hours. Both floating and attached cells were harvested. After counting the cell number, cells were plated in non-coated 60 mm tissue culture dishes at 10^3 ^cells/ml, 5 ml/dish in LHC-9/RPMI. Cells were maintained in culture for 7–10 days with medium changes every 2–3 days. Cells were then fixed with PROTOCOL (Fisher Diagnostics. Middletown, VA) and photographed. All colonies present in each well, defined as a cluster of 20 or more cells, were counted visually.

### Statistical analysis

All data are expressed as mean ± standard error of the mean. Statistical comparisons of multi-group data were analyzed by analysis of variance (ANOVA) followed by student's *t *test for values that appeared different with the Tukey's (one-way) or Bonferroni's (two-way) post-test correction for multiple comparisons using PRISM4 software. P < 0.05 was considered significant.

## Results

**Figure 1:**
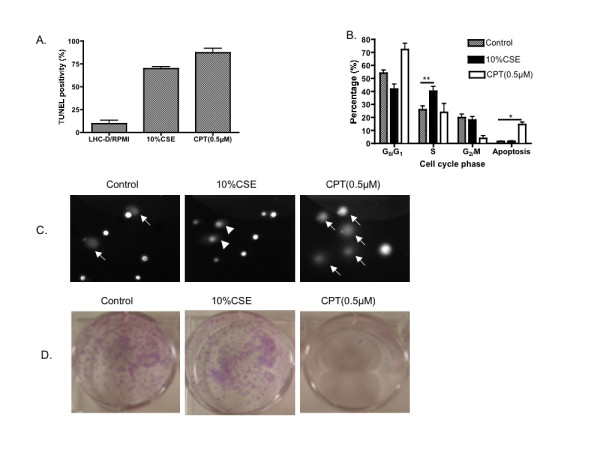
**Effect of cigarette smoke on human bronchial epithelial cell DNA damage and survival**. Human bronchial epithelial cells were exposed to 10% cigarette smoke extract for 24 hours or to 0.5 μM camptothecin for 4 hours, respectively, in LHC-D/RPMI medium. Both floating and attached cells were harvested, combined and used for TUNEL assay (Panel A), DNA content profiling and cell cycle analysis (Panel B), COMET assay (Panel C) and colony formation assay (Panel D). Data presented in panels A, C and D is one representative experiment from at least 4 replicates in both BEAS-2B and HBEC cells. Panel B is an average of 8 different experiments for "control and CSE", and 4 different experiments for camptothecin (CPT) treated. * p < 0.05, ** p < 0.01 compared to control. Panel C: arrow heads indicate cells with DNA damage, and arrows indicate cells undergoing apoptosis with a typical fan-like tail and small head.

*Cigarette smoke extract induces DNA damage without leading to apoptosis *Cigarette smoke induced DNA damage in human bronchial epithelial cells as evidenced by TUNEL positivity (Figure 1A). To further determine if apoptosis occurred in these DNA-damaged cells, confluent HBECs were treated with 10% CSE or 0.5 μM camptothecin. DNA content by FACS analysis and DNA damage/apoptosis by COMET assay were then evaluated. Compared to medium alone (control), 10% cigarette smoke extract did not increase apoptosis in human bronchial epithelial cells as evaluated by either DNA content or COMET assay (Figure 1B and 1C). In contrast, camptothecin induced apoptosis in these cells as evidenced by a sub-G1 peak (14.6 ± 1.8%, Figure 1B) or cells with a small DNA head and fan-like tail in the COMET image (apoptotic index: 43.5% ± 8.9%, Figure 1C). Cigarette smoke, however, led to cell cycle arrested in S phase (Figure 1B, 40.2 ± 3.8% vs 25.9 ± 2.9% of control, p < 0.01). Furthermore, cells with DNA damage, if allowed to recover, were able to proliferate and form colonies in subsequent culture (Figure 1D). In addition, necrosis was not induced by cigarette smoke extract (10% or lower) as evidenced by both MTT and LDH assay (data not shown).

### Effect of cigarette smoke on NF-κB activation

**Figure 2:**
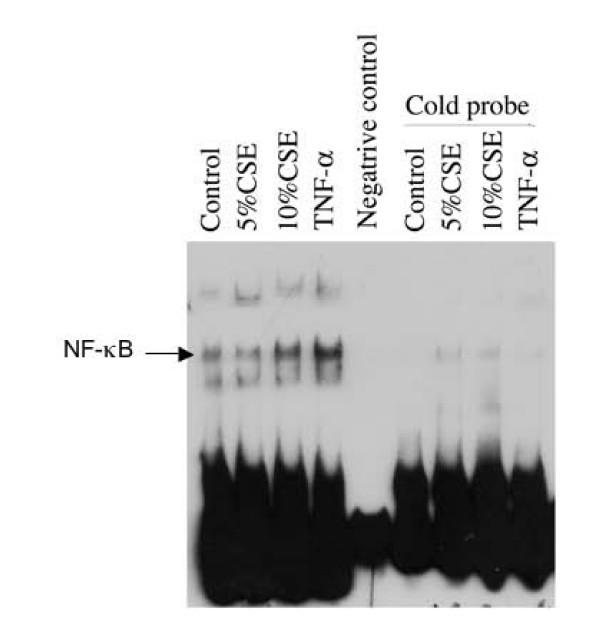
**Effect of cigarette smoke on NF-κB and DNA binding**. Cells were exposed to 0, 5 and 10% CSE or TNF-a (10 ng/ml) for 30 minutes in LHC-D/RPMI medium. Nuclear proteins were extracted and subjected for EMSA as described in the methods. Data presented are one representative of 3 separate experiments with similar results.

Since NF-κB is involved in regulating cell death and survival in variety of cell types, activation of NF-κB in response to cigarette smoke exposure was investigated by EMSA. Cigarette smoke extract (5% and 10%) stimulated DNA binding activity of NF-κB as evidenced by the electrophoresis mobility shift assay (Figure 2).

### Role of NF-κB in modulating cell survival in response to cigarette smoke exposure

**Figure 3:**
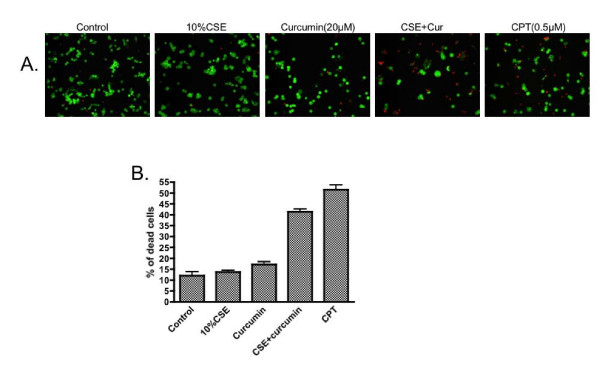
**Effect of NF-κB inhibitor on the viability of HBEC exposed to cigarette smoke extract**. Cells were pre-treated with 20 μM curcumin for 30 minutes followed by exposure to 10% cigarette smoke extract for 24 hours in the presence or absence of curcumin. Cell viability and cytotoxicity were determined by LIVE/DEAD assay. Panel A: One representative micro-photograph of LIVE/DEAD staining. Green: live cells; Red: dead cells. Magnification: 200×. Panel B: Quantitative data from 3 separate experiments. Vertical axis: percent of dead cells = red cells/(green cells + read cells) * 100%. Horizontal axis: treatment.

To investigate the role of NF-κB in modulating cell survival, both the pharmacological inhibitor of NF-κB, curcumin, and RNA interference targeting p65 were used in the current study. Neither 10% CSE nor curcumin (up to 20 μM) alone affected cell viability as examined by LIVE/DEAD Cytotoxicity and Viability assay (Figure 3). When cigarette smoke extract and curcumin were added together, however, the number of dead cells was significantly increased (41.4 ± 1.2% of CSE plus curcumin vs 11.9 ± 1.8% of control, p < 0.01, Figure 3B).

**Figure 4:**
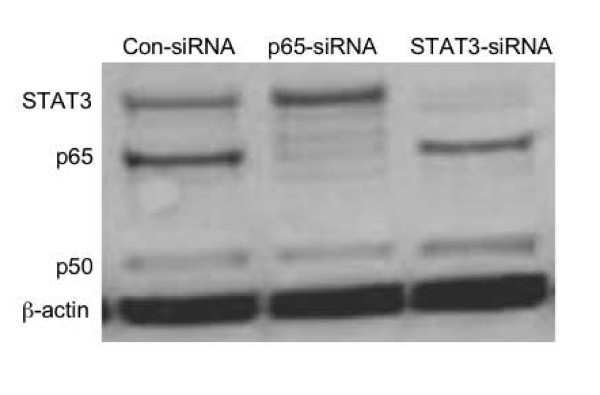
**Specific suppression of p65 by siRNA**. HBECs were transfected with control-siRNA, p65-siRNA or STAT3-siRNA for 6 hours as described in the methods. Cells were maintained in culture for additional 72 hours. Cell lysates were then subjected to immunoblot for p65, p50, STAT3 and β-actin. Data presented are one representative of 4 separate experiments.

The role of NF-κB in mediating cell survival was further investigated by small RNA inference of p65. Following transfection with p65-siRNA, the expression of p65 was suppressed nearly 100%; in contrast, there was little effect on p50 or β-actin, but an apparent increase in expression of STAT3, which was specifically suppressed by STAT3 siRNA (Figure 4).

**Figure 5:**
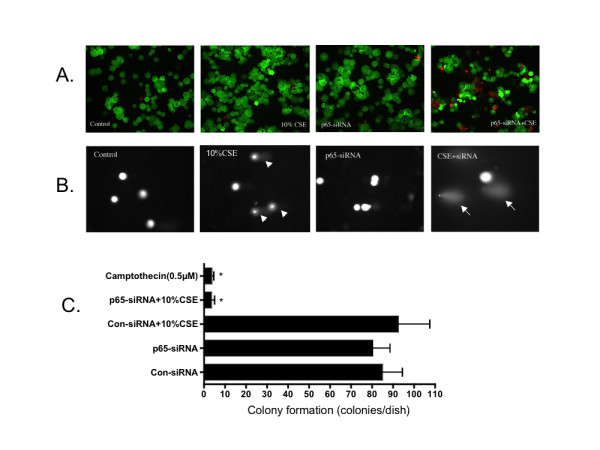
**Role of p65 in mediating HBEC survival in response to cigarette smoke exposure**. Following transfection with p65-siRNA, HBECs were exposed to 10% CSE for 24 hours. Cells were then harvested and used for LIVE/DEAD cytotoxicity/viability assay (Panel A), COMET assay (Panel B) and colony formation assay (Panel C) as described in the methods. Data in panel A and B are one representative from 3 separate experiments, and panel C is an average of 3 separate experiments each performed in triplicate dishes. Panel B: arrow heads indicate cells with DNA damage, and arrows indicated cells undergoing apoptosis.

Following transfection with p65-siRNA, HBEC cells were exposed to cigarette smoke extract for 24 hours. Cell death or survival was then evaluated by three different methods including LIVE/DEAD staining, COMET assay and clonogenic assay. Only the combination of p65-siRNA suppression and smoke exposure resulted in major cytotoxicity. In the presence of 10%CSE, the cells lacking p65 started to detach at 3–4 hrs after exposure to cigarette smoke and nearly all of the cells were detached from the culture dish after 24 hours exposure. In contrast, only a few cells in the control-siRNA treated group detached in response to cigarette smoke exposure. LIVE/DEAD staining demonstrated that very few cells were dead (red nuclei) in the non-targeting siRNA transfected cells (Figure 5A, 3.5 ± 0.3%), in the cells of p65-siRNA transfected without cigarette smoke exposure (Figure 5A, 7.0 ± 1.1%) or in non-targeting siRNA transfected cells exposed to 10% cigarette smoke (Figure 5A, 2.4 ± 1.0%). In contrast, nearly half of the cells were dead in the cells transfected with p65-siRNA and exposed to cigarette smoke extract (Figure 5A, 32.5 ± 1.8%, p < 0.01 compared to control siRNA transfected cells without exposure to cigarette smoke extract), which was similar to the cells exposed to camptothecin (data not shown). Consistent with LIVE/DEAD cytotoxicity assay, p65-depleted cells underwent severe DNA damage and apoptotic death when they were exposed to cigarette smoke as evidenced by COMET assay (Figure 5B). The apoptotic index was significantly higher in the cells lacking p65 and exposed to cigarette smoke (42.5 ± 6.5% vs 6.3 ± 1.2% of control, p < 0.01).

To further evaluate cell survival and ability to proliferate, clonogenic assay was performed. As shown in Figure 5C, neither cigarette smoke exposure of the non-targeting siRNA transfected cells (92.6 ± 14.9 colonies/dish) nor the p65-siRNA transfection alone (80.6 ± 8.1 colonies/dish) altered the ability of the cells to form colonies. However, cigarette smoke exposure of the p65-suppressed cells resulted in a significant decrease in colony formation (3.4 ± 1.4 colonies/dish, p < 0.01 compared to non-targeting siRNA transfection without cigarette smoke exposure 85.6 ± 9.6 colonies/dish). Similarly, colony formation was significantly decreased in camptothecin-treated cells (3.6 ± 1.1 colonies/dish, p < 0.01 compared to non-targeting siRNA transfection without cigarette smoke exposure).

### Role of NF-κB in regulating anti-apoptotic protein Bcl-XL

**Figure 6:**
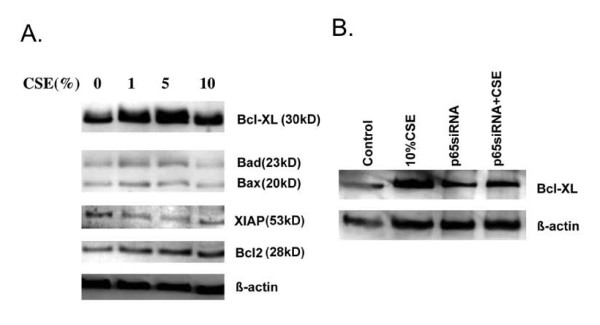
**Role of p65 in regulating anti-apoptotic protein level in response to cigarette smoke exposure. **Panel A: effect of cigarette smoke on the levels of anti-apoptotic and pro-apoptotic proteins. HBECs were treated with varying concentrations of CSE for 24 hours. Cell lysates were subjected for immunoblotting of Bcl-XL, Bad, Bax, XIAP, Bcl2 and β-actin as described in the methods. Panel B: Role p65 in regulating Bcl-XL synthesis in response to cigarette smoke exposure. HBECs were transfected with control siRNA or p65 siRNA followed by exposing to cigarette smoke. Cell lysates were obtained 24 hours after CSE exposure and subjected for immunoblotting for Bcl-XL and β-actin as described in the methods. Data presented is one representative from 2 separate experiments.

To explore the mechanism of NF-κB prevent HBEC from death in response to cigarette smoke exposure, effect of cigarette smoke on anti-apoptotic protein synthesis was investigated. As shown in Figure 6A, cigarette smoke significantly stimulated anti-apoptotic protein Bcl-XL with strongest effect at 5%CSE. CSE also slightly stimulated Bcl2, another anti-apoptotic protein, but did not affect levels of pro-apoptotic proteins, Bax and Bad. Furthermore, up-regulation of Bcl-XL by CSE in HBECs was significantly reduced in the cells lacking of p65 (Figure 6B).

## Discussion

The current study demonstrated that cigarette smoke extract activates NF-κB as evidenced by DNA binding activity, and by this mechanism, blocks cell death following DNA damage in response to cigarette smoke exposure in human bronchial epithelial cells. The inhibition of NF-κB activity by a pharmacologic inhibitor (curcumin) or suppression of p65 by siRNA results in a significant increase in apoptotic cell death in response to cigarette smoke exposure, indicating NF-κB regulates cell survival of human bronchial epithelial cells following cigarette smoke-induced DNA damage.

Cigarette smoke contains over six thousand chemical compounds that are delivered to the lungs as a gas, as an aerosol or as particles. Many of these toxins can cause DNA damage or strand breakage. In response to DNA damage, cells can either repair the damage or can protect the integrity of the genome by activation of pathways leading to programmed cell death, i.e. apoptosis [15,16]. Should apoptosis fail to remove cells with irreversibly damaged DNA, this may result in epigenetic changes or somatic cell mutation, which could lead to altered tissue function and, potentially, to the development of cancer or other disease. Here, we report that cigarette smoke induces not only DNA damage, but also cell cycle arrest in S phase. These cells, however, did not undergo apoptosis. In contrast, the cells exposed to cigarette smoke recovered following removal of the cigarette smoke and could proliferate and form colonies. Similar findings have been reported by Bayram et al in diesel exhaust particle exposed human lung epithelial cells and by Narayan et al in cigarette smoke exposed human breast epithelial cells *in vitro *[17,18].

Cell cycle arrest allows more time for DNA repair following DNA damage. Timely progression of cell cycles is regulated by a series of specific check-point kinase (CHK1, CHK2), cyclin-dependent kinases (cdks) and their inhibitors (cdkIs) [19,20]. The most potent cdkIs are p21, p27, p57 and p16 [21]. Activation of these cdkIs leads to G1/S phase cell cycle arrest in response to DNA damage [22-24]. In contrast, suppression or deficiency of p21 protein leads the cells to undergo apoptosis through extensive cytochrome c release and caspase activation in response to DNA damage [25,26]. It has been reported that cells with activated NF-kB in response to DNA damage had prolonged cell cycle arrest time followed by cell survival [27]. In contrast, cells that failed to activate NF-kB underwent transient cell cycle arrest and extensive cell death [27]. Furthermore, induction of p21 in the arrested cells was NF-kB dependent and suppression of p21 by siRNA reduced NF-kB-mediated cell survival [27]. However, whether p21 also regulates cell cycle arrest in human bronchial epithelial cells in response to cigarette smoke-induced DNA damage remains to be determined.

Whether a cell undergoes apoptosis or survival following DNA damage is controlled by a complex interaction of many signaling pathways [28-31]. Among these, NF-κB is believed to play an important role in regulating cell survival by up-regulating anti-apoptotic proteins [27,32-34]. NF-κB is a family of transcription factors that can form either homo- or heterodimers. Five distinct chains (RelA (p65), RelB, cRel, p50/p105, p52/100) comprising NF-κB have been described, providing considerable heterogeneity. Latent forms are retained in the cytoplasm bound to a class of inhibitory proteins termed IκBs [35]. A large number of stimuli are capable of activating a family of kinases termed IκB kinase (IKK), which phosphorylates IκB, and leads to its degradation. This results in unbound NF-κB that is then free to enter the nucleus to modulate gene expression. Many inflammatory stimuli and injurious insults, including cigarette smoke, can activate NF-κB [7,36-38]. Consistent with previous reports [7,8,39,40], we found that cigarette smoke extract stimulates NF-κB DNA binding activity in human bronchial epithelial cells.

We have previously reported that cigarette smoke extract induces DNA damage without leading to apoptosis [2], and that the IL-6/STAT3 pathway plays a role in mediating cell survival in response to cigarette smoke exposure [13]. Here, we extend these previous studies by demonstrating that inhibition of NF-κB signaling by curcumin or suppression of p65 by siRNA also results in increased cell death of bronchial epithelial cells in response to cigarette smoke extract. This indicates that NF-κB is also involved in regulating cell survival following cigarette smoke-induced DNA damage. The downstream mechanisms by which NF-κB mediates cell survival remains to be further defined. However, like IL-6/STAT3 [13], NF-κB can modulate the production of a number of pro- and anti-apoptotic proteins, especially, Bcl-XL. This suggests that multiple pathways that may interact by regulating the network of proteins that in turn modulate apoptosis are required to sustain cell survival in the face of DNA damage induced by cigarette smoke.

In the current study, the pharmacologic inhibitor curcumin was used to inhibit NF-κB signaling. Curcumin [1,7-bis-(4-hydroxy-3-methoxyphenyl)-1,6-heptadiene-3,5-dione] is the major bioactive compound in turmeric (Curcuma longa) with reported antioxidant, anti-inflammatory, anti-carcinogenic, and anti-mutagenic effects [41,42]. Inhibition of NF-κB activity by curcumin sensitizes cancer cells to chemotherapy, inhibits cell growth, and induces apoptosis in cancer cells [43,44]. Consistent with this, our data demonstrate that curcumin sensitizes bronchial epithelial cells to undergo apoptotic death in response to a low concentration of cigarette smoke extract that would otherwise cause DNA damage without cell death and that this effect is dependent on NF-κB activity.

Curcumin, however, inhibits not only NF-κB, but also other transcription factors including AP-1 and STATs, and kinases such as protein kinases and MAPKs [45-47]. Therefore, in addition to curcumin, an RNAi strategy specifically targeting p65 was also used in the current study. Suppression of p65 by RNAi has been reported previously [48,49]. In the current study, we demonstrated that p65-siRNA very efficiently and specifically suppressed p65 production without affecting non-targeted STAT3, p50 and β-actin in human bronchial epithelial cells. Suppression of p65 by RNAi *per se *does not alter cell viability or the ability of cells to proliferate. Cell death only occurred when the cells lacking p65 were exposed to cigarette smoke, indicating p65 is required for protection of the cells from cigarette smoke insult.

It has been reported that NF-kB modulates cell survival or death in many cell types through regulating expression of apoptosis associated proteins [34,50-52]. Constitutive activation of NF-kB contributes to the survival of cancer cells while inhibition of NF-kB sensitizing the cells to undergo apoptosis in response to chemotherapy or radiotherapy [53-55]. In the current study, we demonstrated that an anti-apoptotic protein, Bcl-XL, was significantly up-regulated by cigarette smoke in HBECs and this was abolished by introduction of p65 siRNA prior to cigarette smoke exposure. These results suggest that NF-kB regulates HBEC survival, at least in part, through up-regulating the anti-apoptotic protein, Bcl-XL. Cell survival, however, depends on a balance of many factors, and it seems likely that NF-kB will modulate factors other than Bcl-XL.

Effect of cigarette smoke on human being is determined by many factors such as type of cigarette, manner of smoking and history of smoking. In the current study, an *in vitro *cell culture model was used to study the effect of cigarette smoke on human airway cells. This *in vitro *cell culture model has its limitations as it differs from *in vivo *conditions. In this study, the cells were in submerged culture condition and were exposed to CSE in tissue culture media without protein supplementation. It is likely that the *in vivo *exposure of cells to toxins in inhaled smoke will differ, both due to the different concentrations of toxins and to the modulatory effects of proteins and other factors present in the cellular milieu. Despite these limitations, however, *in vitro *experiments with the design utilized in the current study have a long history of providing useful information [56,57]. In this regard, the calculated concentration of acrolein in our fresh medium (2.16 × 10–5 M) is similar to that reported by others [58] and to that inhibits lung fibroblasts repair functions [59]. Furthermore, the key *in vitro *findings of the current study, that NF-κB mediates bronchial epithelial cell survival following DNA damage by cigarette smoke exposure provides evidence for a potential mechanism by which cigarette smoke may cause epigenetic changes or somatic cell mutation.

## Conclusion

The current study demonstrates that cigarette smoke extract not only induces DNA damage and cell cycle arrest without leading to apoptosis, but also stimulates NF-κB -DNA binding activity and up-regulates Bcl-XL, and that NF-kB is required for CSE induced inhibition of apoptosis through Bcl-XL up-regulation. By damaging DNA and simultaneously inhibiting apoptosis through NF-kB signaling pathway, cigarette smoke may be able to cause either epigenetic changes or somatic cell mutation. Either of these could lead to altered cellular function and could contribute to chronic diseases such as cancer and chronic obstructive pulmonary disease.

## Abbreviations

CPT: Camptothecin; CSE: Cigarette smoke extract; DMSO: dimethyl sulfoxide; HBEC: human bronchial epithelial cells; TUNEL: terminal dUTP-biotin nick-end labeling

## Competing interests

The authors declare that they have no competing interests.

## Authors' contributions

XL, SR- design experiment, write and revise manuscript. PB-interpretation of the results. ST, MM, HK, QF, TK, XW, LM-carry out cell culture, cigarette smoke extract preparation and cell exposure, immunoblot and apoptosis assays. All authors read and approved the final manuscript.
